# Development of Low Phytate Rice by RNAi Mediated Seed-Specific Silencing of Inositol 1,3,4,5,6-Pentakisphosphate 2-Kinase Gene (*IPK1*)

**DOI:** 10.1371/journal.pone.0068161

**Published:** 2013-07-02

**Authors:** Nusrat Ali, Soumitra Paul, Dipak Gayen, Sailendra Nath Sarkar, Karabi Datta, Swapan K. Datta

**Affiliations:** 1 Plant Molecular Biology and Biotechnology Laboratory, Department of Botany, University of Calcutta, Kolkata, West Bengal, India; 2 Division of Crop Science, Indian Council of Agricultural Research (ICAR), New Delhi, India; University of Delhi South Campus, India

## Abstract

Phytic acid (InsP_6_) is considered to be the major source of phosphorus and inositol phosphates in most cereal grains. However, InsP_6_ is not utilized efficiently by monogastric animals due to lack of phytase enzyme. Furthermore, due to its ability to chelate mineral cations, phytic acid is considered to be an antinutrient that renders these minerals unavailable for absorption. In view of these facts, reducing the phytic acid content in cereal grains is a desired goal for the genetic improvement of several crops. In the present study, we report the RNAi-mediated seed-specific silencing (using the *Oleosin18* promoter) of the *IPK1* gene, which catalyzes the last step of phytic acid biosynthesis in rice. The presence of the transgene cassette in the resulting transgenic plants was confirmed by molecular analysis, indicating the stable integration of the transgene. The subsequent T_4_ transgenic seeds revealed 3.85-fold down-regulation in *IPK1* transcripts, which correlated to a significant reduction in phytate levels and a concomitant increase in the amount of inorganic phosphate (Pi). The low-phytate rice seeds also accumulated 1.8-fold more iron in the endosperm due to the decreased phytic acid levels. No negative effects were observed on seed germination or in any of the agronomic traits examined. The results provide evidence that silencing of *IPK1* gene can mediate a substantial reduction in seed phytate levels without hampering the growth and development of transgenic rice plants.

## Introduction

Phytic acid (*myo*-inositol-1,2,3,4,5,6-hexakisphosphate or IP_6_) is known as the major source of phosphorus in cereal grains, comprising approximately 1–2% of the dry weight and accounting for approximately 65–80% of the total seed phosphorus [1]. In most cereals, with the exception of maize (*Zea mays*), approximately 80% of the total phytic acid (IP_6_) accumulates in the aleurone layer of the grains. In general, IP_6_ accumulates in the protein storage bodies as mixed salts called phytate that chelate a number of mineral cations. During the process of germination, endogenous grain phytase is activated, which degrades phytate, releasing stored phosphorus, *myo*-inositol and bound mineral cations [1] that are further utilized by the developing seedlings. However, due to the lack of microbial phytase enzymes [2], monogastric animals are unable to remove the phosphates from the *myo*-inositol ring and are, therefore, incapable of utilizing the phosphorus present in cereals [3]. Phytate has six negatively charged ions, making it a potent chelator of such divalent cations as Fe^2+^, Zn^2+^, Ca^2+^, and Mg^2+^ and rendering these ions unavailable for absorption by monogastric animals [4]. In view of these adverse effects, many attempts have been made to reduce the phytic acid content in cereals.

Among the different approaches for reducing the phytate levels in cereals, the exogenous expression of recombinant microbial phytase is common [5], [6], [7], [8], and another promising strategy is the generation of cereal mutants exhibiting a low phytic acid (*lpa*) phenotype [4]. Several *lpa* mutant lines have been generated in rice [9], [10], wheat [11], and maize [1], [12], [13]; although effective, these strategies are sometimes associated with downstream impacts on crop yield and other parameters of agronomic performance [4]. Therefore, a different strategy was pursued, whereby transgenic crops were developed by manipulating the phytic acid biosynthetic pathway [3], [14]. Recently, twelve genes from rice (*Oryza sativa* L.) have been identified that catalyze intermediate steps of inositol phosphate metabolism in seeds [15].

The first step of phytic acid biosynthesis in the developing rice seed is catalyzed by *myo-*inositol-3-phosphate synthase (MIPS, EC 5.5.1.4) [16], and various attempts have been made to silence the expression of the *myo*-inositol-3-phosphate synthase (*MIPS*) gene under the control of constitutive [17] or different rice seed-specific promoters [3], [14]. In the case of constitutive promoters (*CaMV35S*), expression of the *MIPS* gene was also suppressed in vegetative tissues in addition to the seeds, causing detrimental effects to the plant. Hence, seed-specific promoters, e.g., *GlutelinB-1* (*GluB-1*) and *Oleosin18* (*Ole18*), have been used to mediate suppression only in the seeds. The resulting transgenic rice plants exhibited more pronounced silencing with the *Ole18* promoter, as it drives expression specifically in the aleurone layer and embryo of seeds [18], the site of maximum phytate accumulation. However, the inadvertent change in seed *myo*-inositol content was not considered and might have a negative impact on plant inositol metabolism, as *myo*-inositol-3-phosphate, the product of MIPS, is known to be the only precursor for the *de novo* synthesis of *myo*-inositol [19], [20], [21]. Therefore, to reduce the phytate content in seeds without disturbing related pathways, enzymes involved at a later stage in phytic acid biosynthesis (i.e., IPK; Inositol phosphate kinases) in rice should be targeted and the effects analyzed.

IPK1 (Inositol 1,3,4,5,6-pentakisphosphate 2-kinase) is believed to catalyze the final step in phytic acid biosynthesis, whereby the InsP_5_ molecule is phosphorylated at the 2^nd^ position [22], [23], [24]. The InsP_6_ biosynthetic pathway was previously described in *Saccharomyces cerevisiae* [24], and the pathway was found to share a common final step with that in *Dictyostelium discoideum*: the phosphorylation of Ins (1,3,4,5,6)P_5_ to InsP_6_ by a 2-kinase enzyme designated as IPK1 (EC 2.7.1.158). The *S. cerevisiae IPK1*Δ mutant showed an almost complete inability to synthesize InsP_6_ and showed a reduction in the ability to export mRNA from the nucleus. Several *myo*-inositol kinase enzymes have since been identified in plants, including *myo*-inositol kinase [13], Ins(1,3,4)P_3_5/6-kinase [25], Ins(1,4,5)P_3_6/3/5-kinase, and Ins (1,3,4,5,6) P_5_ 2-kinase [26], [27]. Recent reports examined the Ins(1,4,5)P_3_6/3/5-kinase (*AtIpk2*β*-1*) and Ins(1,3,4,5,6)P_5_ 2-kinase (*AtIpk1-1*) genes using T-DNA insertion mutants in *Arabidopsis* [28], and the phytate content was reduced in the *AtIpk2*β-*1* mutant by 35% in the *AtIpk1-1* by 83% and by more than 95% in the double mutant.

In the present study, we generated transgenic rice plants by silencing the last step of phytic acid biosynthesis in the Pusa Sugandhi II rice cultivar by manipulating the expression of the *IPK1* gene using a seed-specific promoter, *Ole18*, in an RNAi-mediated approach. The resulting T_4_ transgenic plants were analyzed at the molecular and biochemical levels, revealing substantial reductions in phytate levels and an increase in the amount of inorganic phosphate (Pi). In addition, we also estimated the change in the concentration of different metals in the rice grains after milling, as metal ions may be affected by reduced seed phytate levels. Different agronomic traits of the transgenic plants were also analyzed and compared with non-transgenic rice plants.

## Materials and Methods

### Plant Material and Growth Conditions

*Oryza sativa* L. subspecies *indica* cv. Swarna and IR-36, procured from Chinsurah Rice Research Station, Hooghly, West-Bengal, were used for cloning purposes. For purposes of genetic transformation, *Oryza sativa* L. subspecies *indica* cv. Pusa Sugandhi II was obtained from IARI, ICAR, India. Following surface sterilization, the seeds were germinated on distilled water-soaked filter paper in a plant growth chamber (FLI-2000, Eyela, Japan) maintained at 30°C and 75% relative humidity.

Low-phytate transgenic (T_0_–T_4_) rice generated and respective non-transgenic control (non-transformed Pusa Sugandhi II rice) plants were grown in pots containing fertilizer-enriched paddy field soil (N: P: K = 80∶40: 40 kg/ha) under greenhouse conditions. The day/night temperature regime of 30/25°C under condition of natural illumination, and a relative humidity of 70–80% was maintained throughout the experiment.

### Construction of Plasmids

Total RNA was extracted from *indica* rice cultivar using the RNeasy Plant mini kit following the manufacturer’s protocol (Qiagen). After RNA quantification, cDNA was synthesized from the purified RNA using the Superscript III reverse transcriptase two-step RT-PCR kit (Invitrogen, USA). The RT-PCR product of the *IPK1* (GenBank accession no. AK102842; LOC_Os04g56580) gene amplified using gene-specific primers (*IPK1*F, 5′-CTGCTCTTCTAA TTTCTGACC-3′, and *IPK1*R, 5′-CTTCTTAATGTTTGTCTACTG-3′) was purified and cloned into the pENTR-D TOPO entry vector (Invitrogen) and sequenced. The 1.1-kb fragment of the *IPK1* gene from the entry clone (pENTR-*IPK1*) was then introduced into the binary destination vector pIPKb006 using an LR clonase (Invitrogen, USA) based recombination reaction [29]. Lastly, the *Ole18* promoter (GenBank accession no. AF019212) cloned from the IR36 rice cultivar using a specific primer pair (*Ole18*F, 5′-TCAGCCAATACATTGATCCG-3′, and *Ole18*R, 5′-GCAAGATGAATGCAACGAAG-3′) was ligated to the MCS of the recombined vector at the *Spe*I and *Hind*III sites. The complete RNAi vector (p*Ole18*-*IPK1*-006) containing the *IPK1* gene under the control of the *Ole18* promoter was used for the rice transformation experiments.

### Genetic Transformation and Selection of Transgenic Plants

Biolistic transformation was performed following the protocol described in previous reports [30]. Immature embryos of *indica* rice cultivar Pusa Sugandhi II were used for genetic transformation of the prepared plant transformation vector construct (p*Ole18*-*IPK1*-006) with a particle delivery system (PDS-1000/He system, BIORAD, Hercules, CA, USA) following the manufacturer’s instructions. Following bombardment, the immature embryos were transferred to callus induction medium (MS with 30 g L^−1^ sucrose, 2 mg L^−1^ 2, 4-D, and 8 g L^−1^ agar) supplemented with 50 mg L^−1^ hygromycin B for selection and maintained in the dark at 27°C for 45 days. The tissue was passed through three successive selection cycles of two weeks each. Hygromycin-resistant embryogenic calli were selected and transferred to regeneration medium (MS with 30 g L^−1^ sucrose, 2 mg L^−1^ kinetin, 0.5 mg L^−1^ NAA, and 8 g L^−1^ agar) and maintained under a 16/8-hour photoperiod at 28°C for 20 days. The regenerated plants were then transferred to rooting medium (MS without hormone) for 15 days. After the development of a proper root system, individual plants were transferred to the greenhouse and grown to maturity. All the plants were fertile and exhibited a normal phenotype.

### Southern Hybridization

Genomic DNA was isolated from positive T_4_ transgenic plants and non-transgenic control plants using the DNeasy Plant mini kit following the manufacturer’s protocol (Qiagen). The DNA was quantified using a Nanodrop spectrophotometer (Thermo Fisher, USA), and Southern hybridization was performed according to a standard protocol [31]. Genomic DNA (10 µg) was digested separately with *EcoR*I and *Hind*III (Fermentas), separated on a 1% agarose gel, and transferred to a nylon membrane (Hybond N+, Amersham, GE Healthcare). The *RGA2* intron (PCR product) labeled with [α-^32^P]-dCTP radioisotope (BARC, India) using the Decalabel DNA labeling kit (Fermentas) was used as the probe for hybridization. X-ray film was exposed in the dark at −80°C.

### Quantitative RT-PCR Analysis

Total RNA was isolated from mature, dehusked T_4_ seeds using a TRIZOL (Invitrogen, USA)-based modified RNA isolation protocol [32]. The purified RNA was treated with DNase (Roche, USA) to eliminate genomic DNA contamination. First-strand cDNA was synthesized using 4 µg of total RNA and the transcriptor high-fidelity cDNA synthesis kit (Roche, USA) following the manufacturer’s instructions. The qRT-PCR reaction was performed in triplicate in 96-well optical plates using gene-specific primer pairs (In*IPK1*F, 5′-TGAGAAGATTGTCAGGGACTT TC-3′, and In*IPK1*R, 5′-CGTACTCAGAATCTGTTGTTCCA-3′; In*IPK2*F, 5′-GATTAAACG GTCCAACAT-3′, and In*IPK2*R, 5′-GGTATCAGTTGCCGTAAG-3′; and In*ITP5/6K*F, 5′-GAT TTGCATACAGGCGACAA-3′, and In*ITP5/6K*R, 5′-ATCGCAAGCAGTTCCACAA-3′) and SYBR Green (Fermentas). The optimized cycle (40 cycles) was as follows: 95°C for 30 s, T_m_°C for 30 s, and 72°C for 30 s. The procedure was according to the manufacturer’s instructions (CFX 96 Real time system, Bio-Rad). The quantitative variation was evaluated in different samples using the ΔΔCt method, and the amplification of the *β-tubulin* gene (*tubulin*F, 5′-ATG CGTGAGATTCTTCACATCC-3′, and *tubulin*R, 5′-TGGGTACTCTTCACGGATCTTAG-3′) was used as the internal control to normalize all the data.

### Determination of Seed Phosphorus Levels

The total phosphorus in the seeds was extracted using the alkaline peroxodisulfate digestion method [33]. An equal number of individual seed samples (transgenic and non-transgenic) was crushed, and 2 mL of digestion reagent (0.27 M potassium peroxodisulfate/0.24 M sodium hydroxide) and 10 mL of deionized water were added. The sample mixture was autoclaved at 120°C for 60 min. A 1-mL aliquot of the extract of each sample was centrifuged at 20,000 × g for 10 min, followed by spectrophotometric assay at 800 nm [34].

For the analysis of the inorganic phosphate (Pi) levels, individual seed sample of T_4_ transgenic and non-transgenic control were ground to powder. The crushed powder was further extracted with 12.5% (w/v) trichloroacetic acid containing 25 mM MgCl_2_ and centrifuged at 20,000×g for 10 min. The supernatant was collected, and the Pi level was determined using 4 mL of freshly prepared Chen’s reagent (6 N H_2_SO_4_, 2.5% ammonium molybdate, and 10% ascorbic acid). The absorbance of the resulting colored complex was measured at 800 nm [34].

### Phytic Acid Content Analysis by HPLC

HPLC analysis of phytic acid was based on metal replacement reaction of the phytic acid from colored complex of iron (III)–thiocyanate and the decrease in concentration of the colored complex was monitored [35]. Prior to extraction, each sample (transgenic and non-transgenic control seeds) was homogenized well in a mortar using a pestle. A 200 mg of each sample was weighed and extracted with 0.5 M HCl by continuous stirring for 1 hr at RT followed by centrifugation at 4000 rpm for 15 min. The supernatant was collected and stored at 4°C for further use. For HPLC analysis, 0.1 mL of sample extract was placed in a 3-mL glass tube and mixed with 0.9 mL ultra-pure water and 2 mL of iron (III)–thiocyanate complex solution. The mixture was stirred in a 40°C water bath for 2.5 hr and cooled at room temperature. After centrifuging the mixture for 5 min, 20 µL of the supernatant was injected onto the column of a reverse-phase HPLC system (Waters, USA). The mobile phase was a mixture of 30% acetonitrile in water including 0.1 M HNO_3_, and the flow was adjusted to 1 mL min^−1^. The peak of iron (III)–thiocyanate was detected at 460 nm. The phytic acid concentration was calculated using the calibration curve prepared with a phytic acid standard (Sigma Aldrich; P0109).

### Analysis of Seed *myo*-inositol Content

Non-transgenic control and T_4_ transgenic seeds were ground to powder and extracted with 10 volumes of 50% aqueous ethanol. The *myo*-inositol derivative was prepared by dissolving the residue in 50 µL of pyridine and 50 µL of trimethylsilylimidazole: trimethylchlorosilane (100∶1). Following incubation at 60°C for 15 min, 1 ml of 2,2,4-trimethylpentane and 0.5 mL of distilled water were added. The sample was then vortexed and centrifuged for 5 min, and the upper organic layer was transferred to a 2-mL glass vial [21]. The *myo*-inositol content was quantified as a hexa-trimethylsilyl ether derivative by GC-MS (Trace GC Ultra, Thermo Scientific). The samples were injected in the split mode (split ratio 10) with the injector temperature at 250°C and the oven at 70°C. The oven temperature was ramped at 25°C min^−1^ to 170°C after 2 min, continuing at 5°C min^−1^ to 215°C, increased at 25°C min^−1^ to 250°C, and reverted to the initial temperature. The electron impact mass spectra from m/z 50–500 were attained at −70 eV after a 5-min solvent delay [21]. *Myo*-inositol hexa-trimethylsilyl ether was identified using the database library NIST07 (MS Library Software) by comparing the mass fragmentation pattern. At the same time *myo*-inositol standards (Sigma Aldrich; 57569) in aqueous solution were dried, derivatized, and analyzed.

### Quantification of Metals

An equal number of mature T_4_ transgenic and non-transgenic seeds were dehusked and then milled in a rice miller (Satake, Japan) for 30 s. The milled seeds were weighed (2 g) and then digested using a modified protocol of dry-ashing digestion [36]. The acidic ash solution was filtered through Whatman no. 42, and the final volume was brought up to 25 mL. The metal content (i.e., Ca, Fe, Zn, and Mg) of the sample extract (clear filtrate) was analyzed using an Atomic Absorption Spectrometer (AAS, AAnalyst 200, Perkin Elmer, USA) with respective hollow cathode lamps (HCLs, Perkin Elmer).

### Amino Acid Analysis

The amino acid analysis of the rice samples (transgenic and non-transgenic control seeds) was according to the AccQ-tag method following the manufacturer’s instructions (Waters, USA)**.** Approximately 20 mg of ground rice powder from each sample was digested with 2 mL of 6 N HCl containing 0.1% phenol at 110°C for 16 hours. The digested rice samples were filtered through 0.22-µM filters (Millipore), and the filtrate was neutralized with freshly prepared 6 M NaOH solution. The neutralized samples and diluted amino acid standard (10 µL) were derivatized using the AccQ-Fluor reagent kit (WAT052880-Waters Corporation, Milton, MA, USA) according to the manufacturer’s protocol. The AccQ-Fluor amino acid derivatives were separated on a Waters 2695 Separations Module HPLC System attached to a Waters 2996 fluorescence detector. A 10-µL aliquot of the sample was injected onto a Waters AccQ-Tag Column (150 mm×3.9 mm). The mobile phase was a mixture of Waters AccQ Tag Eluent A diluted (1∶10, Eluent A, WAT052890) and 60% acetonitrile (Eluent B) in a separation gradient according to the manufacturer’s protocol.

### Activity of Enzymes during Seed Germination

The activity of α-amylase [37], β-amylase [37], [38], and α-glucosidase [39] were analyzed at different time periods after germination in non-transgenic and transgenic seeds.

#### a. α-Amylase assay

Germinating seeds were collected at 0, 12, 24, 36, 48, 60, 72, 84, and 96 hours and stored frozen at −80°C. The seed samples of both transgenic and non-transgenic control plants were crushed in 50 mM phosphate buffer (pH 7.0) and centrifuged at 4°C for 15 min. The supernatant was collected, and the enzyme assay was performed by incubating 100 µL of the enzyme extract with 1 mL of soluble starch (1%) at 50°C for 15 min. The reducing sugar released was estimated by the addition of the dinitrosalicylic acid (DNS) reagent [40].

#### b. β-Amylase assay

β-amylase was measured following a reported protocol [37]. The seeds were homogenized with 4 mL ice-cold 16 mM sodium acetate buffer, pH 4.8. The homogenate was centrifuged at 12,000×g for 15 min, and the supernatant was used for determining the β-amylase activity. A 0.5-mL aliquot of the enzyme extract was added to 0.5 mL of 1% potato starch in 16 mM sodium acetate buffer equilibrated at 37°C for 2 min, vortexed, and incubated with shaking for 5 min at 37°C. A 0.5-mL aliquot of 3,5-dinitrosalicylic acid (DNSA) reagent was added to the reaction mixture and boiled for 5 min. The absorbance at 540 nm was measured after adding 4.5 mL distilled water. The DNSA reagent consisted of 1% 3,5-dinitrosalicylic acid, 0.4 M NaOH, and 1 M potassium sodium tartrate. A standard curve using maltose solution was prepared in a similar manner.

#### c. α-Glucosidase assay

The determination of the α-glucosidase activity was performed as per a modified protocol [39], [41]. One gram of finely ground seeds collected at 0, 12, 24, 36, 48, 60, 72, 84, and 96 hours after germination was extracted for crude α-glucosidase by adding 10 mL of 10 mM acetate buffer (pH 5.0, containing 5 mM DTT and 90 mM NaCl). The mixture was maintained at room temperature for 30 min and then centrifuged at 3,000×g at 4°C for 15 min. A*f*ter filtration, the supernatant was assayed for enzyme activity: 100 µL of crude enzyme was mixed with 1 mL of 6 mM p-nitrophenyl-α-D-glucopyranoside (PNPG) in 100 mM acetate buffer, pH 4.5. The reaction was performed at 40°C for 10 min and terminated by adding 0.5 mL of 200 mM Na_2_CO_3_. The amount of p_-_ni_t_rophenol liberated from PNPG was measured using a spectrophotometer at 400 nm. Blanks for the reaction were prepared in the same manner, but 0.5 mL of 200 mM Na_2_CO_3_ was added before mi_x_ing with the crude enzyme.

### Morphological Analysis of Transgenic Plants

#### Seed germination assay

A controlled germination test (CGT) and accelerated ageing test (AAT) were performed to assess the germination capability of the T_4_ transgenic seeds compared to the non-transgenic control [42]. For CGT, the seeds were soaked in water for 8 hr at 30°C and then transferred to fresh water (CGT) at 30°C for an additional 12 hr. The seeds were then rinsed two to three times in distilled water and germinated on filter paper soaked with distilled water at 30°C in the dark. For AAT, seeds were incubated in a growth chamber with 80% relative humidity at 45°C for 48 or 96 hr and then allowed to germinate at 30°C in the dark, as in case of CGT. The germination percentage was recorded at regular intervals and analyzed. The experiment was repeated three times to confirm the observations.

#### Agronomic performance of transgenic plants

The agronomic performance of the transgenic rice plants growing under greenhouse conditions was evaluated with respect to the non-transgenic control. Different agronomic parameters, such as plant height (cm), number of effective tillers, number of panicles per plant, panicle length, 1000 grain weight (DW), seed length, and breadth, were considered during the study. The height of individual plants was measured as the distance from the soil surface to the tip of the panicle of the longest tiller. The panicle length was averaged from 5 randomly selected panicles of each plant. After harvest, up to 100 dried mature grains from each plant were weighed, and the 1000-grain weight was calculated accordingly. Five randomly chosen plants from each transgenic line were evaluated for each parameter studied.

### Statistical Analysis

All of the statistical analyses were performed using the Graph Pad Prism 5 software. The experimental data values are presented as the means ± standard error (SE) based on three to five replications. The means were compared by ANOVA, and the significant differences between group means were calculated following Bonferroni Post-tests.

## Results

### Generation of Transgenic Rice Plants

**Figure 1:**
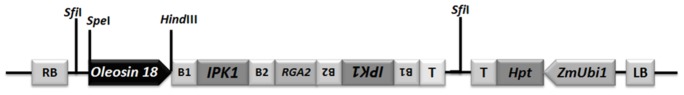
Schematic diagram showing partial map of RNAi vector construct. p*Ole18*-*IPK1*-006 vector construct showing the *IPK1* gene cloned in sense and antisense orientation separated by wheat *RGA2* intron. *HPT* gene was used as the plant selection marker. (T = *CaMV 35S* terminator).

**Figure 2:**
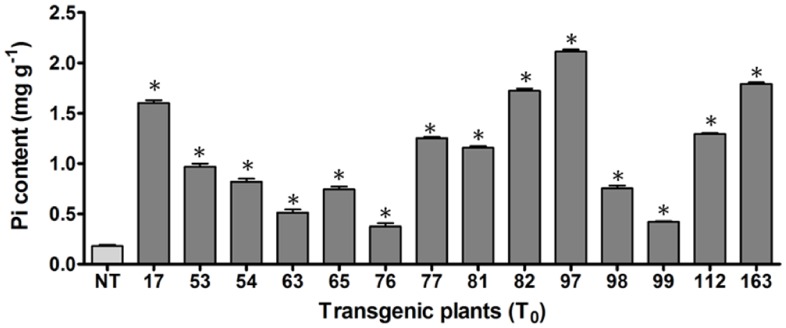
Screening of transgenic plants based on inorganic phosphate (Pi) content. Pi fractions in non-transgenic (NT) and T_0_ transgenic rice plants were analyzed from the seeds. The symbol * indicates significant differences at P = 0.05 (n = 3).

The transgenic plants generated were screened for the presence of the transgene cassette (Fig. 1) by PCR analysis using wheat *RGA2* intron-specific primer pairs (*RGA2*F, 5′-CCTGAAATTGGT AAAAGTAGA-3′, and *RGA2*R, 5′-TGTATCTTCATACTGCATTTG-3′). The genomic DNA from 21-day-old plants showed amplification of the wheat *RGA2* intron only in the transgenic-positive plants, whereas no amplification was observed in the non-transgenic control (non-transformed Pusa Sugandhi II rice cultivar) plants. In the T_0_ generation, forty-five individual putative transgenic rice plants were generated of which approximately thirty were positive for the corresponding transgene, as confirmed by genomic PCR analysis. Among the positive transgenic plants screened, fourteen plants (T_0_) showing higher Pi levels were selected, and the T_1_ generation was produced (Fig. 2). The transgenic plants (T_1_) exhibiting higher Pi levels (IO6–17, 82, 97, 112, and 163) were further selected, and subsequent generations (T_2_–T_3_) were grown under greenhouse conditions until maturity. After successive screening of the consequent generations (T_1_–T_3_), the progeny of IO6-97 (IO6-97-9-4) and IO6-163 (IO6-163-10-5) were selected. In the T_3_ generation, IO6-97-9-4 and IO6-163-10-5 showed a maximum Pi content (data not shown), thus all of the analysis were performed with the progeny of these two transgenic lines in the T_4_ generation.

### Expression Analysis of the Transgenic Plants

**Figure 3:**
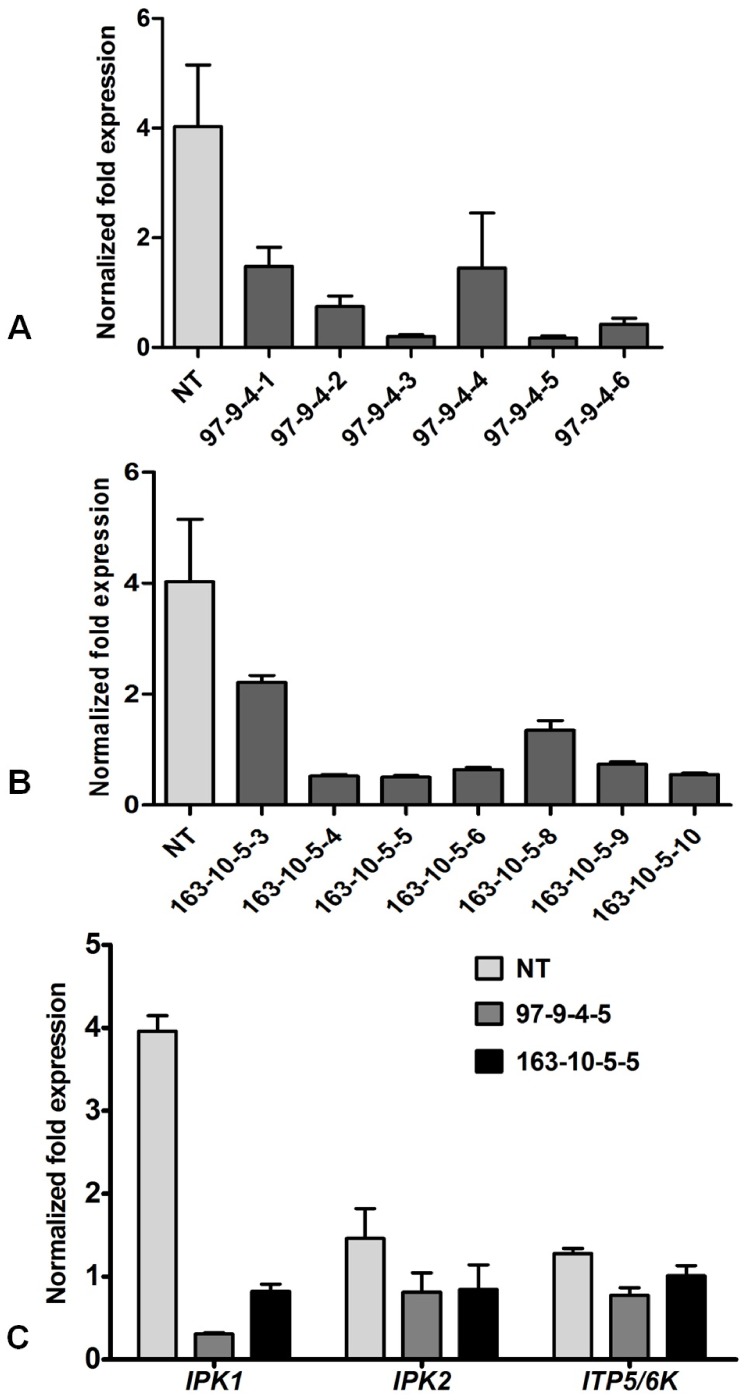
Expression analysis of transgenic rice plants. qRT-PCR analysis of T_4_ transgenic seeds of (A) IO6-97-9-4 and (B) IO6-163-10-5, as compared to the internal control *β tubulin* reveals down-regulation in the transcript level of *IPK1*. The normalized fold expression clearly indicates varied level of silencing, the maximum reduction being 3.85-fold as observed in 97-9-4-5. (C) Expression levels of *IPK1*, *IPK2* and *ITP5/6K* genes in selected RNAi transgenic lines IO6-97-9-4-5 and IO6-163-10-5-5. (NT = Non-transgenic control).

To quantify the level of down-regulation in the transgenic seeds (T_4_), a quantitative real-time PCR analysis was performed using SYBR Green (Fermentas). The *β-tubulin* gene was used as the reference gene to normalize all the data. The normalized fold reduction in the levels of *IPK1* transcripts varied widely among the different progeny of IO6-97-9-4 (Fig. 3A) and IO6-163-10-5 (Fig. 3B). However, a maximum reduction of 3.85-fold was observed in the transgenic line IO6-97-9-4-5 (T_4_), revealing a distinct down-regulation of *IPK1* and suggesting successful silencing mediated by the RNAi vector construct (p*Ole18*-*IPK1*-006). Furthermore, the gene expression of other inositol phosphate kinase genes, i.e., *IPK2* (inositol 1,4,5-tris-phosphate kinase/inositol polyphosphate kinase) and *ITP5/6K* (inositol 1,3,4-triskisphosphate 5/6-kinase) involved in phytate biosynthesis along with *IPK1*, was analyzed in the selected RNAi transgenic line IO6-97-9-4-5 and IO6-163-10-5-5 (Fig. 3C). The results clearly showed that, although the *IPK1* gene displayed a maximum down-regulation, the expression of *IPK2* and *ITP5/6K* was not significantly affected (P≥0.05).

### Southern Blot Analysis of the Transgenic Plants

**Figure 4:**
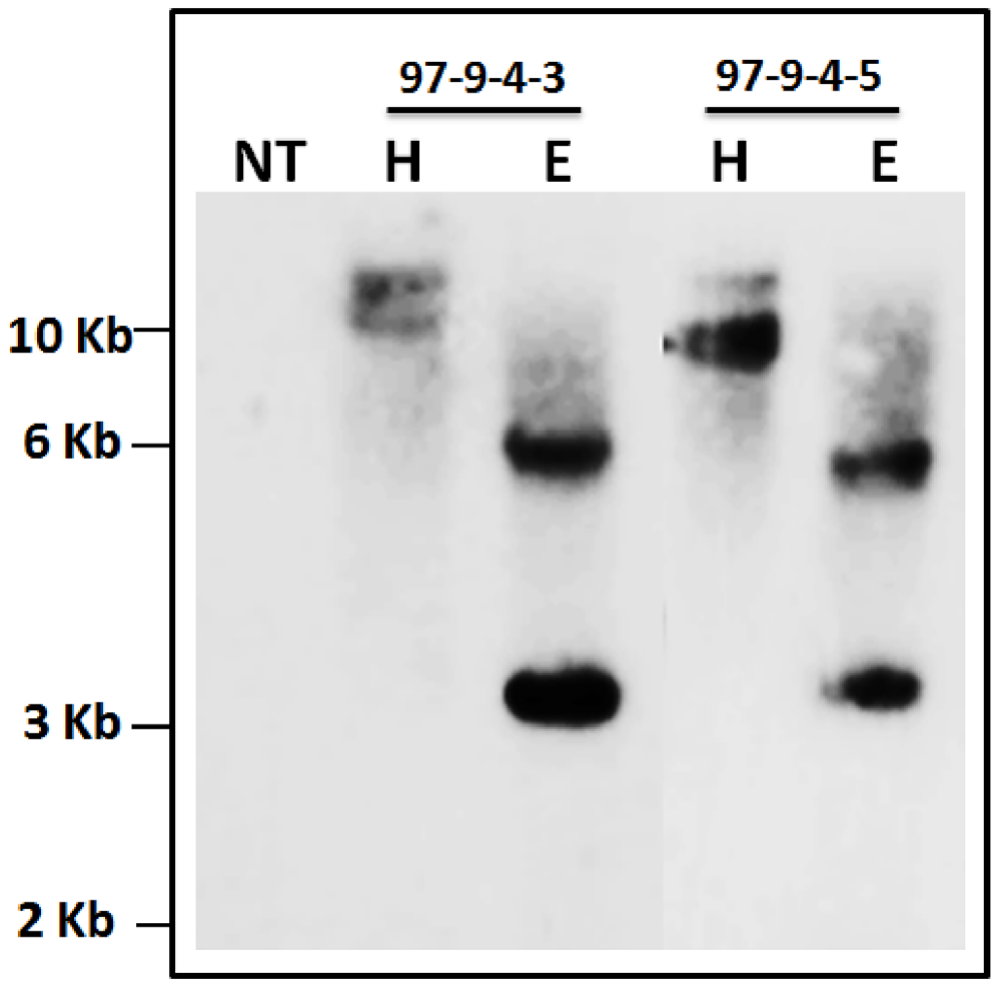
Southern blot analysis of T_4_ progenies of line IO6-97. Stable integration of *RGA2* intron was detected in transgenic rice plants, no hybridization signal was observed in the respective non-transgenic control. Each lane consists of 10 µg genomic DNA, digested with *EcoR*I or *Hind*III. The position and sizes of markers are indicated (NT = Non-transgenic control, E = *EcoR*I and H = *Hind*III).

The integration of the transgene cassette in the genome of line IO6-97 was confirmed by Southern blot analysis using two different restriction enzymes: *EcoR*I and *Hind*III. The results revealed the stable integration of the transgene cassette into the progeny (T_4_). The Southern hybridization pattern showed two bands (in the case of both *EcoR*I and *Hind*III) corresponding to the transgene (*RGA2* intron) in transgenic plants of the examined line. However, no hybridization signal was detected for the non-transgenic control plants (Fig. 4).

### Analysis of Seed Phosphorus and Phytic Acid Levels

**Figure 5:**
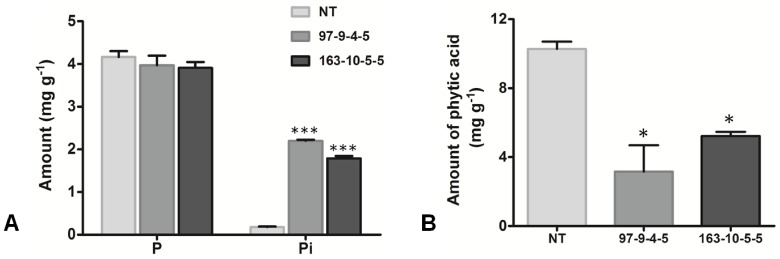
Analysis of Phosphorus and phytic acid content in the transgenic rice seeds. (A) Total phosphorus and Pi content in non-transgenic (NT) and T_4_ low phytate transgenic seeds and (B) amount of phytic acid in non-transgenic (NT) as compared to T_4_ transgenic seeds. The symbols * and *** indicates significant differences at P = 0.05 and 0.001 respectively (n = 3).

The total phosphorus and inorganic Pi levels in the seeds of the non-transgenic rice and T_4_ seeds of transgenic lines IO6-97-9-4-5 and IO6-163-10-5-5 were estimated, and the average total phosphorus content of the T_4_ transgenic and non-transgenic seeds was found to be 3.971 mg g^−1^ (IO6-97-9-4-5), 3.907 mg g^−1^ (IO6-163-10-5-5) and 4.162 mg g^−1^, respectively. No significant difference was observed between the different transgenic sublines and the non-transgenic control seeds (P≥0.05). The Pi levels in the non-transgenic and T_4_ transgenic seeds were analyzed to further determine the storage form of phosphorus in the seeds. The average Pi concentration in the non-transgenic seeds constituted 4.33% of the seed total phosphorus. However, the average Pi content in the IO6-97-9-4-5 and IO6-163-10-5-5 seeds constituted 55.78% and 45.46% of the seed total phosphorus, which was significantly higher than that of the non-transgenic seeds (Fig. 5A). Although the seeds exhibited higher Pi levels, they displayed a normal phenotype, and no aberrations were observed.

HPLC analysis was performed to quantify the phytic acid levels in the seed extracts of the transgenic and non-transgenic control, with the determination of phytic acid based on the replacement of phytic acid with the thiocyanate ligand from the iron (IIII)–thiocyanate complex. The chromatogram obtained from the HPLC/UV-vis method indicated that the transgenic seeds (showing larger peaks of iron (IIII)–thiocyanate complex) had lower phytate levels compared to the respective non-transgenic control, which exhibited a smaller peak, signifying a higher concentration of phytic acid in the seeds. The mean phytic acid values, as calculated from the corresponding peak area, were 10.28 mg g^−1 ^for the non-transgenic seeds, 3.16 mg g^−1^ for the transgenic line IO6-97-9-4-5, and 5.23 mg g^−1 ^for IO6-163-10-5-5 (Fig. 5B). These results represented an average reduction in the seed phytic acid content of 69% for line IO6-97-9-4-5 and approximately 50% for line IO6-163-10-5-5.

### Seed *myo*-inositol Content

**Figure 6:**
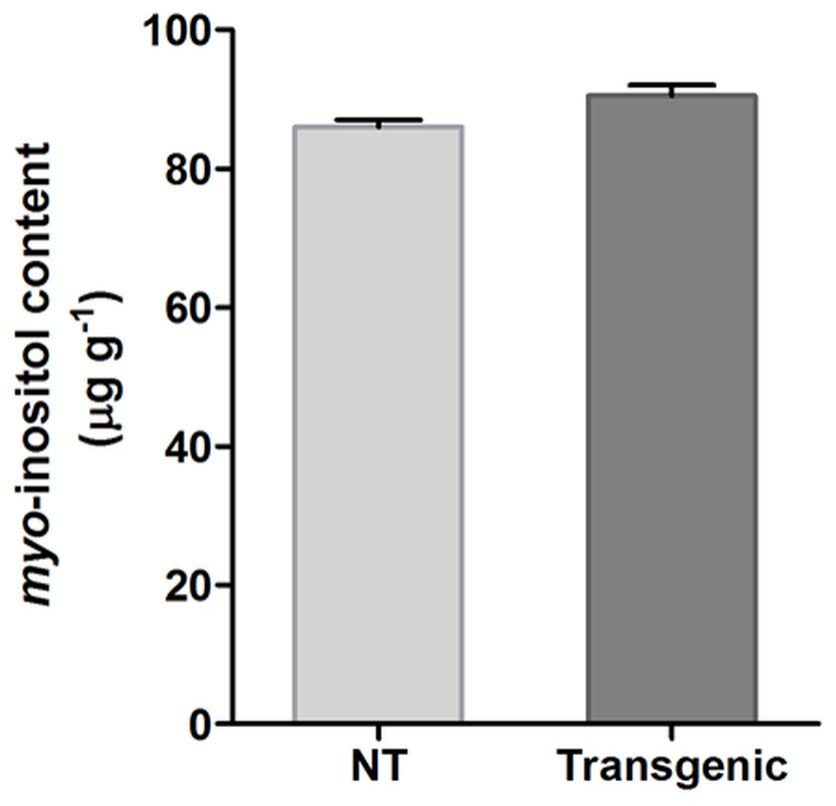
Effect of *IPK1* silencing on seed *myo*-inositol content. *Myo*-inositol content of T_4_ transgenic seeds (IO6-97-9-4-5) as compared to non-transgenic (NT) seeds showed no significant difference (P≥0.05).

It has previously been established that phytic acid biosynthesis is closely related to *myo*-inositol synthesis [43], [21]. Hence, we also examined the effect of silencing the last step of phytate biosynthesis on the seed *myo*-inositol levels. The GC/MS analyses clearly showed that there was no significant difference between the *myo*-inositol content of the transgenic (T_4_) and non-transgenic control seeds (Fig. 6). Therefore, it can be considered that silencing the expression of *IPK1* does not have any effect on the seed *myo*-inositol level, which is desirable because of the important role of *myo*-inositol in plant metabolism and other developmental processes.

### Quantification of Metal Content in Seeds

Phytic acid is known to be a potent chelator of divalent cations and, therefore, renders these metal cations unavailable. Therefore, we examined the level of different metals (Fe, Zn, Ca, and Mg) in the milled seeds of low-phytate T_4_ transgenic seeds compared to that of the non-transgenic control using atomic absorption spectroscopy (AAS, Perkin Elmer). The amount of different metal cations was found to be higher in the low-phytate rice seeds when compared to their respective non-transgenic control (Table 1). Among the different metals analyzed, iron increased to a maximum of 12.61 µg g^−1 ^in the transgenic seeds, whereas the level was 7.03 µg g^−1^ in the non-transgenic control seeds (Table 1). These results indicate a 1.8-fold increase in the levels of iron in milled low-phytate transgenic seeds.

**Table 1:** Metal content as analyzed by Atomic Absorption Spectroscopy from T_4_ milled seeds of greenhouse grown plants.

Metals	Non-transgenic	Transgenic
Calcium (µg g^−1^)	5.32±0.06	7.52±0.08
Iron (µg g^−1^)	7.03±0.07	**12.61±0.22**
Zinc (µg g^−1^)	22.30±0.37	26.62±0.29
Magnesium (mg g^−1^)	0.57±0.01	0.73±0.01

Values are mean ± SE, n = 3.

### Amino Acid Analysis

**Figure 7:**
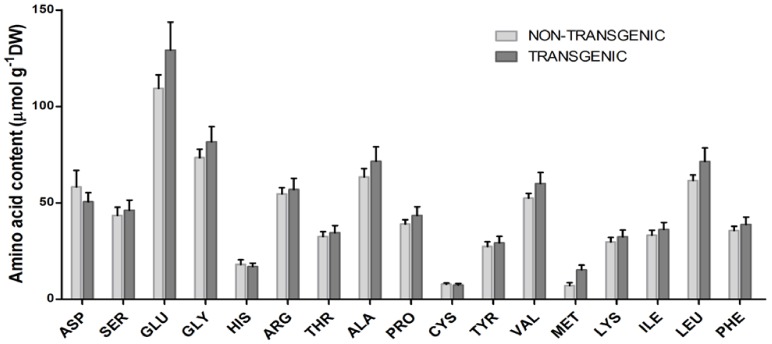
Amino acid analysis in mature grains of non-transgenic and T_4_ transgenic plants. Diagram representing the individual amino acid content of non-transgenic and the transgenic rice grains calculated with respect to the amino acid standard. The error bars indicate SE of three biological replicates for each sample. The data represented here for the transgenics is averaged from the observations of both IO6-97-9-4-5 and IO6-163-10-5-5.

To assess the effect of silencing *IPK1* on the different seed storage proteins of rice, we quantified the individual amino acids by HPLC analysis using the AccQ-tag method. The results showed no significant difference (P≥0.05) between the amount of individual amino acids analyzed from the seeds of the T_4_ transgenic (IO6-97-9-4-5 and IO6-163-10-5-5) and non-transgenic control plants (Fig. 7). Therefore, it can be suggested that the seed-specific suppression of *IPK1* did not lead to any deleterious effects on other seed storage proteins.

### Seed Germination Analysis

**Figure 8:**
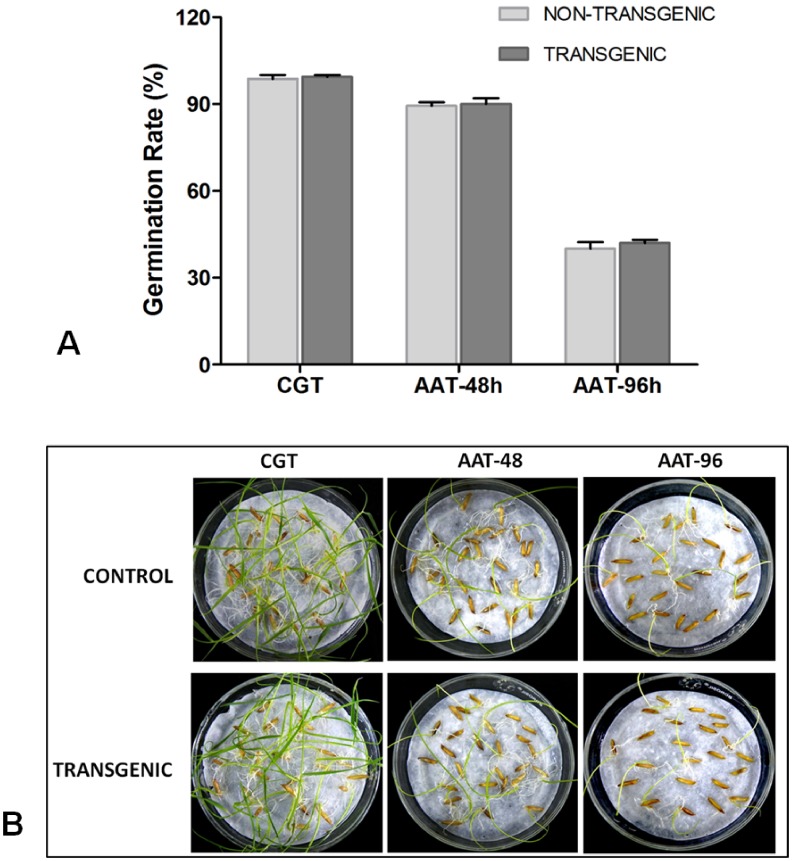
Analysis of seed germination potential in non-transgenic and T_4_ transgenic low phytate seeds. (A) Rate of germination as observed during control germination test (CGT) and accelerated ageing test (AAT) in both non-transgenic and the transgenic rice seeds. (B) Picture showing the morphology of transgenic seeds with respect to the non-transgenic control as recorded at 8th day of germination during the CGT and AAT.

**Figure 9:**
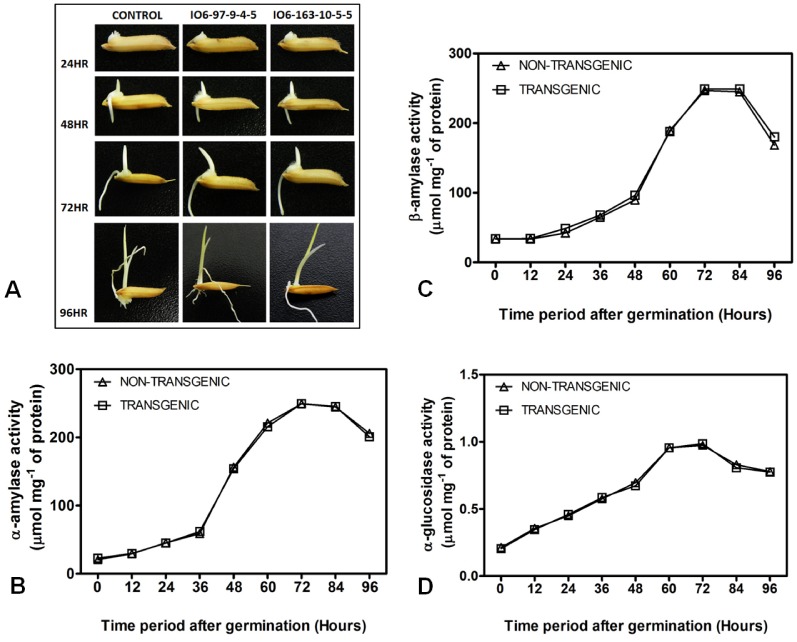
Enzyme activity analysis during germination in T_4_ transgenic and non-transgenic control seeds. (A) Picture showing the phenotype of the seeds during the course of germination at different time intervals. (B) α-amylase, (C) β-amylase and (D) α-glucosidase enzyme activity analyzed at different time intervals after germination in non-transgenic and the transgenic seeds showing no significant differences (P≥0.05). The open triangles represent response of non-transgenic (NT) and opened squares represent response of transgenics. (The data represented here for the transgenics is averaged from the observations of both IO6-97-9-4-5 and IO6-163-10-5-5).

A reduction of phytate levels is often correlated to pleiotropic effects that frequently affect seed development and germination. However, no abnormality was observed at any stage of development or germination in the transgenic seeds with low phytate levels. We also examined the seed germination potential by performing both a control germination test and accelerated ageing test. The germination rate of both the transgenic (T_4_) and non-transgenic control seeds were recorded at regular intervals (Fig. 8A), and the morphological analysis of seed germination revealed similar phenotypes in CGT and AAT of both the transgenic and control seeds (Fig. 8B). In addition to this, the activities of the important starch-degrading enzymes involved in seed germination under optimum conditions were also analyzed (Fig. 9A). Both the transgenic and non-transgenic seeds exhibited similar activities of α-amylase (Fig. 9B), β-amylase (Fig. 9C), and α-glucosidase (Fig. 9D), giving a clear indication that the down-regulation of phytic acid did not interfere with seed germination in the transgenic plants.

### Morphological Traits of Transgenic Plants

The agronomic performance of the T_3_ transgenic plants was compared with that of the non-transgenic plants (Table 2), and different morphological traits were considered and evaluated to analyze the phenotypic alterations in the transgenic plants. All the T_3_ and non-transgenic plants showed similar morphologies at the juvenile stage, and no significant difference was observed in the plant height, number of tillers, and panicles between mature T_3_ and non-transgenic plants (P≥0.05). In addition, the weight of 1000 dry seeds of the transgenic plants was not significantly different from that of the non-transgenic seeds (P≥0.05). All other morphological parameters considered were similar in both the T_3_ transgenic and non-transgenic plants.

**Table 2:** Different parameters considered for agronomic evaluation of T_3_ transgenic plants grown in greenhouse.

Parameters	Non-transgenic control	IO6-97-9-4	IO6-163-10-5
**Plant Height (cm)**	126.0±2.08	124.7±2.02	128.0±1.53
**No. of Tillers**	12.67±0.33	11.67±0.67	12.67±0.67
**No. of Effective Tillers**	11.33±1.20	11.00±0.58	11.67±0.88
**Panicle Length (cm)**	26.00±0.57	24.67±0.88	25.33±0.33
**Grains/Panicle**	73.33±1.76	74.67±0.88	72.33±2.19
**Seed length (mm)**	11.67±0.33	11.50±0.29	11.67±0.33
**Seed breadth (mm)**	2.61±0.01	2.60±0.01	2.62±0.01
**Seed length/breadth ratio**	4.46±0.14	4.40±0.10	4.46±0.14
**1000 seeds dry wt (gm)**	26.67±0.88	25.67±1.20	26.00±0.58

Values are mean ± SE, n = 5.

## Discussion

In the present investigation, we demonstrated the efficient down-regulation of phytic acid, as mediated by silencing of the *IPK1* gene, the product of which catalyzes the last step of the phytate biosynthesis. The enzyme inositol 1,3,4,5,6-pentakisphosphate 2-kinase (IPK1) has been well characterized from different plants, including *Arabidopsis* [27] and maize [44]. Furthermore, in *Arabidopsis*, a T-DNA disruption of the gene for InsP_5_ 2-kinase has been reported to have resulted in an 83% decrease in phytic acid levels [28]. A recent study identified the different enzymes involved in the phytic acid biosynthetic pathway, which included a *IPK1* rice homolog (AK102842) that had 67% similarity to *AtIPK1* [15]. Hence, we generated low-phytate transgenic rice by silencing the gene expression of *IPK1* using an RNAi-mediated approach. It has been established that phytate globoids occur mainly in the aleurone layer of cereals [45], [46] and that the constitutive suppression of enzymes involved in phytate biosynthesis could be detrimental for plant growth and development [17]. Therefore, we used the *Ole18* promoter [14], [18] which has specific activity in the aleurone layer and embryo, to induce the suppression of *IPK1* in rice seeds. The transgenic plants showed the stable integration of the transgene cassette and displayed a normal phenotype. The quantitative RT-PCR analysis of different biosynthetic enzymes (IPK2, ITP5/6K, and IPK1) clearly showed that *IPK1* gene expression was strongly affected in the transgenic rice seeds. The expression analysis of transgenic seeds further revealed a 3.85-fold reduction in the expression of *IPK1* with respect to the non-transgenic control, suggesting efficient silencing of the gene (*IPK1*). In the T_4_ generation seeds of IO6-97, the Pi levels constituted 55.78% of the total seed phosphorus, which was 51% higher than that of the non-transgenic seeds, and the phytic acid content was reduced by approximately 69% with respect to the non-transgenic control. These results give a clear indication that the suppression of *IPK1* actually led to a substantial reduction in phytic acid levels, with a concomitant increase in the amount of Pi.

Based on previous reports, low phytate is often associated with undesirable effects on seed development and germination potential [14], [47], [48]. Therefore, we analyzed the seed germination of the low-phytate transgenic seeds under optimum conditions during control germination tests [42]; the results indicated that the transgenic seeds are viable, showing normal germination patterns with respect to the non-transgenic seeds. It was also noteworthy that the germination potential decreased in a similar fashion, even during the accelerated ageing test in which the seeds of both the transgenic and non-transgenic control plants were subjected to an artificial ageing treatment. The analysis provided a clear idea that, although there is a substantial reduction in the phytate level, this did not interfere with either seed development or subsequent germination under both optimum and stressed conditions. To further verify that the germination process was not impaired, we measured the activity of different starch-degrading enzymes, i.e., α-amylase, β-amylase, and α-glucosidase, which are also known as indicators for assessing germination potential in cereals [49]. All these enzymes showed similar activities in the transgenics when compared to the non-transgenic seeds, indicating normal germination behavior.

The low-phytate transgenic seeds were also analyzed for the content of *myo*-inositol, which is considered to be an important metabolite involved in different biochemical pathways that are further associated with important metabolic processes in plants [50], [51]. In contrast to a previous report that cereal seeds with low phytate showed lower *myo*-inositol levels [21], the T_4_ transgenic seeds of IO6-97 (IO6-97-9-4-5) exhibited a similar *myo*-inositol content as the non-transgenic seeds. Because IPK1 catalyzes the final step of phytic acid biosynthesis [15], it is quite obvious that the silencing of its gene would not affect *myo*-inositol synthesis, which occurs much earlier in the pathway. Therefore, this finding suggests an added advantage for generating low-phytate crops by manipulating *IPK1*, without disturbing the *myo*-inositol levels. In addition to seed *myo*-inositol, which is an essential metabolite playing significant role in different signaling pathways, plant growth and development [51], [52], we also evaluated the individual amino acid content of the transgenic seeds to confirm that the seed-specific manipulation of *IPK1* did not interfere with any of the storage proteins, as cereal seeds are an important source of different seed storage proteins. The results clearly indicated that the amino acid profiles of the transgenic plants were similar to those of the non-transgenic control plants, with no significant differences noted.

It is known that, due to the presence of six negatively charged ions, phytic acid chelates available divalent mineral cations, which renders these minerals less bioavailable [1], [53]. Prior reports have suggested that phytic acid chelates mineral cations and aggregates them as inclusions in protein storage vacuoles (phytate globoids), mainly in the aleurone layer and embryo [4], [54]. Because both the aleurone layer and embryo are removed during commercial milling, a reduced amount of these metals are available in the endosperm, which is generally consumed [55], [56]. In view of these facts, we analyzed the metal concentrations (Ca, Fe, Zn, and Mg) of the low-phytate transgenic seeds (milled) and found a significant increase in metal concentrations in the transgenic compared to the non-transgenic seeds. It was also noteworthy that, among the different metals analyzed, a maximum increase was observed in the levels of iron (Fe), at 1.8-fold more than the non-transgenic control. Although previous reports have suggested an increase in iron content using a dual approach of expressing soybean *ferritin* [57] and *Aspergillus* phytase [5], [8] in cereals, the present investigation highlights that lowering the phytate level alone can lead to elevated iron levels in milled rice seeds.

The strategy undertaken in this research represents a step ahead in developing low-phytate rice, without disturbing the normal behavior of rice plants. The present study confirms that the manipulation of *IPK1* in rice can lead to major reductions in the amount of seed phytate, with a simultaneous increase in the Pi content. Therefore, it can be suggested that the gene encoding IPK1, which catalyzes the last step of phytic acid biosynthesis, is an appropriate candidate to target for reducing phytate levels, without hampering other important metabolic processes and developmental pathways in cereal crops.
